# Interactome of Radiation-Induced microRNA-Predicted Target Genes

**DOI:** 10.1155/2012/569731

**Published:** 2012-06-27

**Authors:** Tenzin W. Lhakhang, M. Ahmad Chaudhry

**Affiliations:** Department of Medical Laboratory and Radiation Sciences, University of Vermont, 302 Rowell Building, Burlington, VT 05405, USA

## Abstract

The microRNAs (miRNAs) function as global negative regulators of gene expression and have been associated with a multitude of biological processes. The dysfunction of the microRNAome has been linked to various diseases including cancer. Our laboratory recently reported modulation in the expression of miRNA in a variety of cell types exposed to ionizing radiation (IR). To further understand miRNA role in IR-induced stress pathways, we catalogued a set of common miRNAs modulated in various irradiated cell lines and generated a list of predicted target genes. Using advanced bioinformatics tools we identified cellular pathways where miRNA predicted target genes function. The miRNA-targeted genes were found to play key roles in previously identified IR stress pathways such as cell cycle, p53 pathway, TGF-beta pathway, ubiquitin-mediated proteolysis, focal adhesion pathway, MAPK signaling, thyroid cancer pathway, adherens junction, insulin signaling pathway, oocyte meiosis, regulation of actin cytoskeleton, and renal cell carcinoma pathway. Interestingly, we were able to identify novel targeted pathways that have not been identified in cellular radiation response, such as aldosterone-regulated sodium reabsorption, long-term potentiation, and neutrotrophin signaling pathways. Our analysis indicates that the miRNA interactome in irradiated cells provides a platform for comprehensive modeling of the cellular stress response to IR exposure.

## 1. Introduction

MicroRNAs (miRNAs) are approximately 21 nucleotides in length that do not code for proteins. miRNAs were discovered in 1993 but their significance was not realized until 2000 [1]. miRNAs act as negative regulators of gene expression by mRNA degradation and protein downregulation [2]. miRNA bind to the target mRNA and initiate mRNA degradation. Alternatively miRNAs inhibit the protein machinery from latching on to the mRNA. The interplay between the miRNA and mRNA forms a highly complex regulatory network, mainly because a single miRNA can target hundreds of different mRNA molecules [3]. Higher production of miRNA leads to lower expression levels of its target proteins. The miRNAs are reported to be involved in cell differentiation, metabolic regulation, apoptosis, and many other biological processes [4]. Dysfunction of miRNA has been associated with numerous cancers [5] and alterations in the expression levels or complete deletion of key miRNAs have been reported in tumor cells [6]. 

Cellular stress pathways protect cells from the deleterious effects of genotoxic insult. Ionizing radiation disrupts cellular homeostasis through multiple mechanisms. The cells respond to stress induced by ionizing radiation exposure through complex processes by activating many pathways ranging from DNA damage processing, signal transduction, altered gene expression, cell-cycle arrest, and genomic instability to cell death [7, 8]. The current data suggests that the exposure to radiation provokes cellular responses controlled in part by gene expression networks [7, 9]. miRNAs regulate gene expression and have been shown to control multiple intracellular processes involved in the response to cellular stress [10, 11]. 

Alterations in the miRNA expression levels occur following exposure to ionizing radiation [12–14]. The miRNA expression levels in primary human dermal microvascular endothelial cells (HDMEC) after 2 Gy radiation treatment indicated upregulation of *hsa-let-7g*, *hsa-miR-16*, *hsa-miR-20a*, *hsa-miR-21* and *hsa-miR-29c*, and downregulation of *hsa-miR-18a*, *hsa-miR-125a*, *hsa-miR-127*, *hsa-miR-148b*, *hsa-miR-189*, and *hsa-miR-503* [15]. miRNA profiles of irradiated lung cancer cells indicated that the level of *hsa-let-7g* was higher in radiosensitive cells Caski, NCI-H460 (H460), and ME180 than in radioresistant cells A549, H1299, DLD1 [16]. Changes in expression patterns of *hsa-miR-92b*, *hsa-miR-137*, *hsa-miR-656*, *hsa-miR-558*, *hsa-miR-660*, and *hsa-miR-662* after low (0.1 Gy) and high (2.0 Gy) doses of X-ray in human fibroblasts were observed [17]. The *hsa-let-7* family miRNAs were upregulated in irradiated M059K cells and downregulated in M059J cells. The *hsa-miR-17-3p*, *hsa-miR-17-5p*, *hsa-miR-19a*, *hsa-miR-19b*, *hsa-miR-142-3p*, and *hsa-miR-142-5p* were upregulated in both M059K and M059J cells. [14]. Radiation treatment of prostate cancer cells changed the expression levels of *hsa-miR-521* [18]. The expression profiles of miRNAs in HCT116 human colon carcinoma cells and its p53-null derivative correlated with p53 status [19]. The expression of *hsa-let-7* family miRNAs, which are negative regulators of the rat sarcoma, *RAS* oncogene, was upregulated in irradiated p53 positive TK6 cells but was downregulated in p53 negative WTK1 cells. The *hsa-miR-15a* and *hsa-miR-16* were upregulated in 0.5 Gy-irradiated TK6 cells but were downregulated after a 2 Gy dose of X-rays [13]. The expression levels of *hsa-let-7* family miRNA and miRNA associated with *MYC* translocation were modulated after gamma radiation treatment in Jurkat cells [12]. While many studies have reported dose-dependent changes in the expression profiles of miRNAs in irradiated IM9 human B lymphoblastic cells [20, 21], human lung carcinoma cell line A549 [22], and human fibroblasts [23]; some studies did not observe any significant alterations in miRNA expression in cells treated with gamma-irradiation [24].

We were interested to examine the role of miRNAs in ionizing radiation- (IR-) induced stress pathways. Although miRNAs have been implicated as crucial posttranscriptional gene regulators, their role in the cellular response to IR is not comprehensively examined. We asked the question: can we use microRNAome and their target genes to corroborate previously identified IR responsive pathways? We also argued if the miRNAome would allow us to discover new perspective to radiation exposure. This study was undertaken (1) to assemble miRNA species that are modulated after radiation exposure in many human cell lines, (2) to identify the genes that are targeted by these miRNA using bioinformatics approaches, and (3) to determine the role of miRNA target genes in radiation-induced cellular pathways. 

## 2. Materials and Methods

### 2.1. Selection of IR-Induced miRNA

We collected the data from our published work on radiation-induced miRNA and also searched the PubMed database to collect articles that investigated the modulation of miRNA after IR exposure. The keywords miRNA, microRNA, ionizing radiation and radiation were used in performing the literature search. This search retrieved 236 research articles. PubMed was a preferred choice over the other available article databases such as Web of Science, BIOSIS Previews (http://thomsonreuters.com/) and Science Direct because PubMed provided comprehensive results that overlapped and displayed more articles. The articles were imported into EndNote after determining its relevancy to miRNA and IR topic. The relevant articles extracted from PubMed were subjected to additional refinement. The research studies which were selected, performed ionizing radiation experiments on human cell lines only and recorded the miRNA expression levels. We identified the miRNA species, cell types, type of radiation, radiation dose, and analysis time from these studies. We then assembled a common list of miRNAs that were investigated among a group of cell types.

### 2.2. Generation of IR-Induced miRNA Database

The miRNA expression data extracted from the published research articles was used to assemble a database. Over 1000 records were generated from the data extraction procedure. Microsoft Excel was utilized to tabulate the information from various articles. This “mastersheet” formed the platform for the subsequent analysis. The data was organized in the Excel “mastersheet” with the following headings: cell type, cell line, radiation type, radiation dose (Gy), dose rate (Gy/min), analysis time (hours) after treatment, miRNA species, qualitative miRNA expression from base, qualitative miRNA expression from base (numerical), quantitative miRNA expression (fold change), and data source. The Pivot Table tool of Microsoft Excel was employed to reorganize the master dataset. This “pivot table” was referenced to the original unchanged mastersheet. The pivot table allowed to generate a list of miRNA that were observed to show altered expression in 5 or more cell lines after exposure to ionizing radiation.

### 2.3. Prediction of miRNA Target Genes

The miRNAs of interest were assembled in Microsoft Access database then searched against the mirDB dataset (http://mirdb.org/miRDB/) [25] to predict target genes. Microsoft Access allowed for the quick creation of local database from which high-level queries could be placed. To obtain gene ontology (GO) terms for each target gene, we equipped the Access database with other genomic datasets, such as Entrez Gene, GaRNeT (Genomics and Randomized Trials Network), and KEGG (Kyoto Encyclopedia of Genes and Genomes) Pathways. The Entrez Gene dataset provided information regarding gene symbol and description, which was linked by the Entrez ID. The GaRNeT dataset allowed linking the mirDB dataset to the Entrez Gene dataset.

### 2.4. Identification and Visualization of miRNA-Predicted Genes and Biological Pathways

The output dataset queried through the Access database provided the predicted target genes for the miRNA species of interest. The Entrez ID list was imported in DAVID (Database for Annotation, Visualization andIntegrated Discovery) (http://david.abcc.ncifcrf.gov/), an online database that can link genes to its biological pathways through the KEGG database. This strategy enabled an enriched analysis of genes for GO terms, using the miRNA target genes with respect to the category of biological process. The miRNA-predicted target genes and biological pathways were visualized with Cytoscape software (http://cytoscape.org/). Cytoscape is an open source software package that allows for powerful visual mappings of the provided datasets. Cytoscape provided a visualization that helped connect miRNA to their respective target genes and associated pathways. Cytoscape enabled us to perform more detailed functional analysis to identify miRNA-mediated networks, biological functions and canonical signaling pathways for both miRNAs and the target mRNAs.

## 3. Results and Discussion

Exposure to ionizing radiation induces various physiological responses including DNA repair, cell cycle arrest, signal transduction, cell death, and cell differentiation [7, 8]. It is becoming clear that changes in the expression profile of many genes play a significant role in these processes [7, 9]. The discovery of miRNA and the establishment of their involvement in regulating the gene expression have prompted the need to interrogate their participation in the radiation response. It is not completely understood how miRNAs function in the cellular response to radiation exposure. Our laboratory is engaged in assessing the miRNA responses in irradiated human cells. We have collected data on IR-induced miRNA expression alterations in a variety of cell types [12–14]. We combined our data along with other published data to compile miRNA species that are modulated in a variety of human cell types. It was important to find miRNAs that were analyzed in different studies, because a singular data point would not have allowed for the formation of relationships between different cell types. This dataset was used for the identification of predicted miRNA target genes and the basis of the functional role of miRNAs in the cell. Our goal was to establish a link between the modulation of miRNA after radiation exposure to the stress induced pathways in the cell.

### 3.1. Cell Lines Investigated for IR-Induced miRNA Expression Analysis

A variety of cell lines have been exploited to examine the miRNA expression profile after radiation exposure. The lymphoblast TK6 cell line has normal p53 and was used as a base to compare IR-induced miRNA from p53 negative WTK1 cell line [13]. The M059J and M059K glioma cell lines isolated from the same tumor specimen differ in the DNA-dependent protein kinase activity. M059J lack this kinase whereas M059K express normal levels [26]. The Jurkat and IM9 are both of lymphoid lineages; Jurkat is of T-cell origin and IM9 is of B-cell origin (ATCC; American Type Culture Collection). These cells are prevalent in many research studies because they provide platform on which to study immunological signaling processes and the production of various chemokines [12, 20, 21]. The A549 and HBE135-E6E7 are cancerous cell lines from lung tissue. The cytogenetic data from ATCC indicate that A549 cell line displays a hypotriploid chromosomal expression; a majority of cells having around 66 chromosomes. The radiation-induced miRNA expression profiles differ considerably among all these cell types. We looked for miRNA expression similarities across these cell lines to find common miRNA responses after radiation exposure.  Table 1 shows information regarding some of these cell lines from where the miRNA dataset was selected in this study.

### 3.2. Identification of miRNA Modulated in IR-Exposed Cell Lines

**Figure 1:**
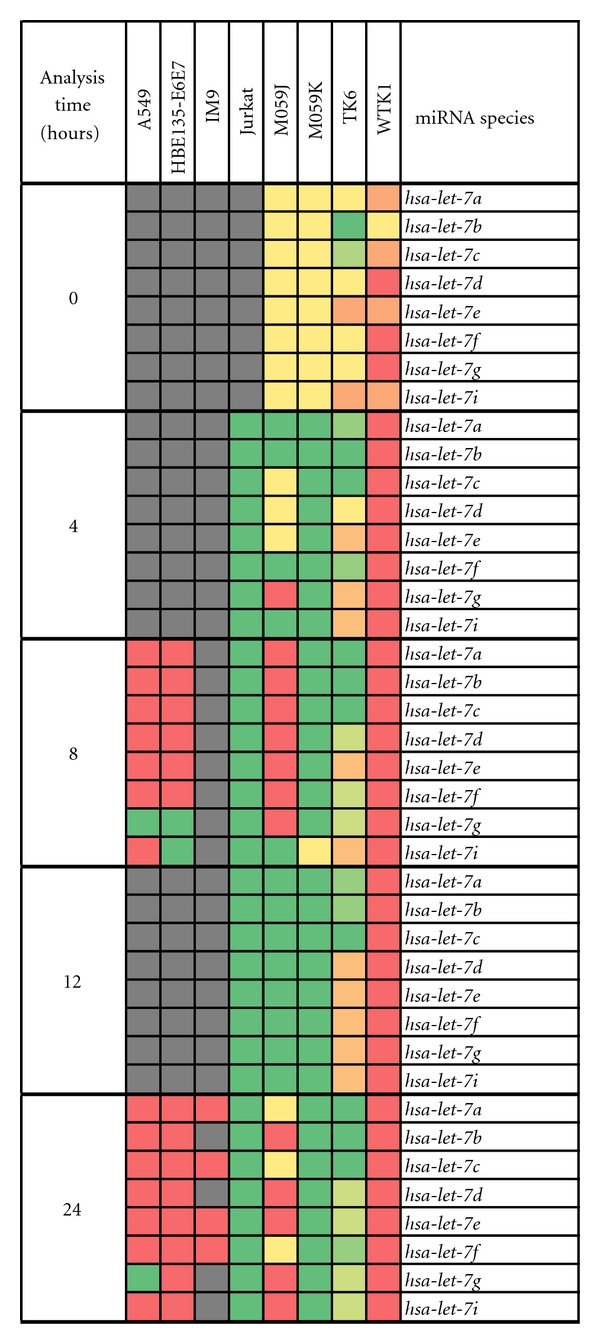
The modulation of *hsa-let-7*-family miRNA in various cell lines. The analysis time indicates the period of time that had elapsed after exposure to ionizing radiation. The green color indicates upregulation and red color shows downregulation of a miRNA. The yellow color signifies no change, and the gray color indicates that the miRNAs during those time points were not examined. The orange color signified some conflicting data where it was either reported a no change or downregulation in two or more studies. Similarly the light green color indicates data where it was either reported a no change or upregulation in two or more studies.

We built an miRNA database to warehouse the IR-induced miRNA expression data extracted from various published studies. All of these studies employed low LET radiation and the doses ranged from 0.1 to 40 Gy. The analysis methodology ranged from real-time PCR to microarray techniques. The number of radiation modulated miRNA ranged from 8 to 36 in individual cell lines. The Microsoft Excel's pivot table from the original “mastersheet” permitted to create a list of miRNA that were modulated in 5 or more cell lines. 20 miRNA species were identified that were modulated in 5 or more cell lines after treatment with IR. The pivot table feature was further exploited to build a heat map of the IR-modulated miRNAs. This heat map displays 3 dimensional information which accounts for cell line, analysis time after IR treatment and the miRNA species (Figure 1). We first looked at the modulation of eight *let-7* family miRNA *hsa-let-7a, hsa-let-7b, hsa-let-7c, hsa-let-7d, hsa-let-7e, hsa-let-7f, hsa-let-7g, *and *hsa-let-7i. *The published studies have examined the expression of these miRNA in various cell lines including A549, HBE135-E6E7, IM9, Jurkat, M059J, M059K, TK6, and WTK1 at 0–24 h time points after exposure to IR. A quick snapshot of the data assembled in Figure 1 indicated that majority of these miRNA were induced in Jurkat, M059J, M059K, and TK6 cell lines. In contrast these miRNA were downregulated in A549, HBE135-E6E7, and WTK1 cells. The expression analysis of *hsa-let-7* family miRNA indicated that both A549 and WTK1 cell lines display similar signatures (Figure 1). The differences in *hsa-let-7* family miRNA expression between eight cell lines revealed that WTK1 displayed marked down regulation in all miRNAs of interest compared to the other cell lines. This could be due to the fact that WTK1 is characterized as an inherently unstable cell line [27]. Even in the absence of any radiation exposure, a high percentage of WTK1 cells was reported to display chromosomal aberrations such as aneuploidy, chromatid breaks, and translocations [27]. These genomic instabilities could be connected to the dysfunction in the microRNAome. Many studies have attempted to probe the role of miRNA in radiation sensitivity. The overexpression or inhibition of let-7g markedly influenced clonogenic survival and cell proliferation; *hsa-let-7g* enhanced radiosensitivity of these cells [15]. Overexpression of *hsa-let-7g* in H1299 cells suppressed the translation of *K-RAS*, and increased the sensitivity to IR. Knockdown of *LIN28B*, an upstream regulator of *hsa-let-7g*, increased the level of mature *hsa-let-7g* and the sensitivity to IR in H1299 cells [16]. The overexpression of *hsa-let-7a* decreased the expression of *K-RAS* and radiosensitized A549 lung carcinoma cells. Inhibition of *LIN28*, a repressor of *hsa-let-7*, attenuated *K-RAS* expression and radiosensitized A549, and pancreatic cancer ASPC1 cells [28]. 

**Figure 2:**
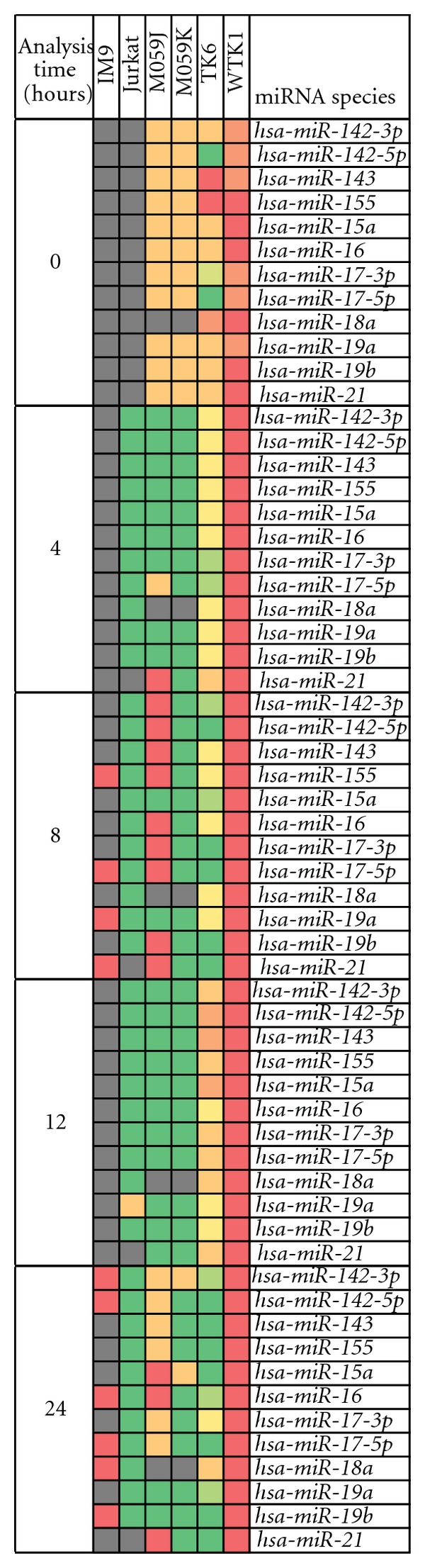
The modulation of various miRNAs in different cell lines. The analysis time indicates the period of time that had elapsed after exposure to ionizing radiation. The green color indicates upregulation and red color shows downregulation of a miRNA. The yellow color signifies no change, and the gray color indicates that the miRNAs during those time points were not examined. The orange color signified some conflicting data where it was either reported a no change or downregulation in two or more studies. Similarly the light green color indicates data where it was either reported a no change or upregulation in two or more studies.

We next determined the expression status of another twelve miRNA hsa-miR-142-3p, hsa-miR-142-5p, hsa-miR-143, hsa-miR-155, hsa-miR-15a, hsa-miR-16, hsa-miR-17-3p, hsa-miR-17-5p, hsa-miR-18, hsa-miR-19a, hsa-miR-19b, and hsa-miR-21 in irradiated IM9, Jurkat, M059J, M059K, TK6, and WTK1 cells (Figure 2). Majority of these miRNA were upregulated in Jurkat, M059J, and M059K cells after exposure to IR but were repressed in irradiated IM9 and WTK1 cells. Some of these IR-induced miRNAs have been implicated in various cancers. In chronic lymphocytic leukemia and prostate cancer, a majority of the patients had deletions or downregulations of hsa-miR-15 and hsa-miR-16 [29, 30]. The hsa-miR-145 and hsa-miR-143 were downregulated in colorectal neoplasia [31]. In lung cancers, miRNA hsa-let-7 was down regulated. The genes that code for miRNAs have been frequently located at genomic locations involved in cancers and there is strong indication that miRNA gene acts as both tumor suppressors and oncogenes [32]. 

### 3.3. Prediction and Visualization of IR-Modulated miRNA Target Genes

**Figure 3:**
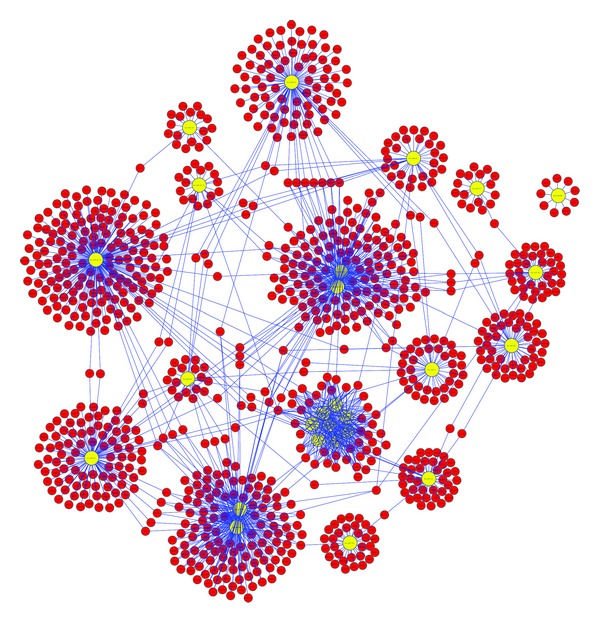
Visualization of miRNAs and their associated target genes network with Cytoscape. The interaction network shows nodes and connections between miRNAs and the target genes. The yellow nodes represent the miRNA and the pink/red nodes represent its targeted gene. The opacity of the blue edge links signifies the target score between the miRNA and the target gene. The opacity of red nodes represents genes that have been identified to play roles in biological pathways.

The list of miRNAs studied among a group of cell types was further examined to identify the target mRNAs affected by these miRNA. We utilized the miRDB for predicting the miRNA target genes. The miRDB uses a support vector machine (SVM), which is a type of algorithm based on statistical learning theory. This program uses statistics and employs artificial intelligence strategy such as neural networks to assign a prediction score for a miRNA's target gene. This process of predicting miRNA target genes is accomplished through a machine-learning algorithm, which places a target score on a miRNA-gene association. This miRDB database was chosen to identify miRNA target genes because for the simplicity of the database structure and agreed upon thresholds for the prediction target score. The prediction target score of 50–95 was used to filter the target genes. We constructed association networks between miRNAs and the target mRNAs and visualized the target genes controlled by various IR responsive miRNA with Cytoscape. The complexity associated with miRNA interactome is shown in Figure 3. As expected a singular miRNA was found to affect the regulation of hundreds of genes and a number of miRNAs were seen to work synergistically with each other to downregulate a multitude of genes. The resulting miRNA : mRNA association network provided nodes and connections between many miRNAs and the target mRNAs (Figure 3). This network demonstrated the overlapping mRNA targets for miRNAs in the *hsa-miR-let7* family, *hsa-miR-15a*, *hsa-miR-16*, *hsa-miR-18a*, *hsa-miR-19a*, *hsa-miR-19b*, *hsa-miR-21*, *hsa-miR-34a*, *hsa-miR-34b*, *hsa-miR-142-3p*, *hsa-miR-142-5p*, *hsa-miR-143*, *hsa-miR-145*, *hsa-miR-155*, *hsa-miR-197*, *hsa-miR-202*, *hsa-miR-376a*, *hsa-miR-575*, and *hsa-miR-609* (Figure 3). Most of the radiation-modulated miRNAs have a large number of mRNA targets. For example, the number of targets genes that were identified in biological pathways for *hsa-miR-15a* and *hsa-miR-16* were 22 (Table 2). The target genes for all these miRNA are shown in Table 2. These interactions demonstrated that a singular miRNA has the capability to affect the regulation of a large number of genes. It was apparent that a number of miRNAs could work together to downregulate several genes.

**Figure 4:**
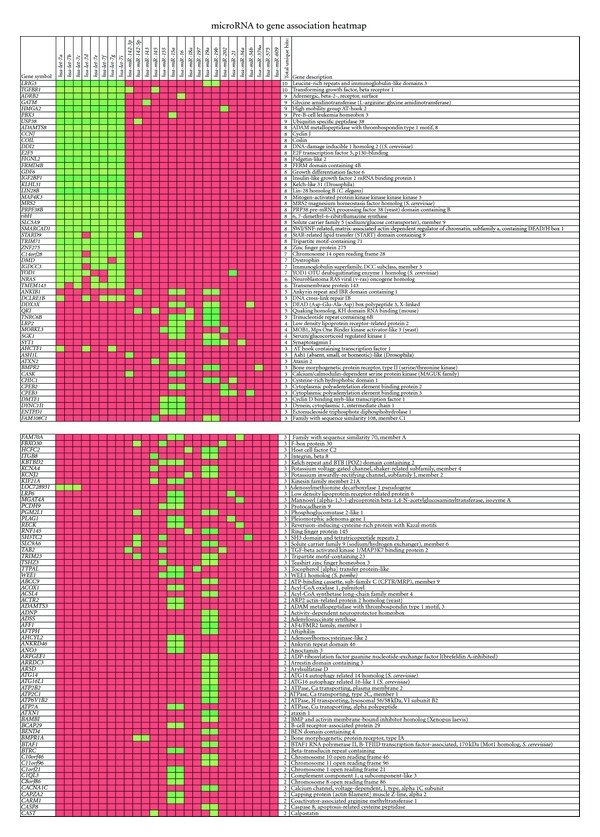
The regulation of genes with various miRNA. The genes that are controlled by two or more different miRNAs are shown. The green shade identifies the miRNA predicted to regulate that particular gene. The red shade identifies the miRNA that are not predicted to control the expression of the listed gene.

We also identified the genes that were controlled by multiple miRNA after exposure to IR.  Figure 4 shows the list of genes along with the miRNA that regulate their expression. The genes that were regulated by two or more miRNA were included in the Figure 4. For example *LRIG3* gene was targeted by *hsa-let-7a, hsa-let-7b, hsa-let-7c, hsa-let-7d, hsa-let-7e, hsa-let-7f, hsa-let-7g, hsa-let-*7*i*, *hsa-miR-19a*, and *hsa-miR-19b*. Similarly *TGFBR1* gene was regulated by *hsa-let-7a, hsa-let-7b, hsa-let-7c, hsa-let-7d, hsa-let-7e, hsa-let-7f, hsa-let-7g, hsa-let-*7*i*, *hsa-miR-142-3p*, and* hsa-miR-145*. This analysis clearly indicated that individual genes were controlled by multiple miRNA after exposure to IR. The net effect of miRNA modulation in irradiated cells is enormous impacting many cellular functions.

### 3.4. Mapping of miRNA-Predicted Target Genes to Biological Pathways Affected by Radiation

**Figure 5:**
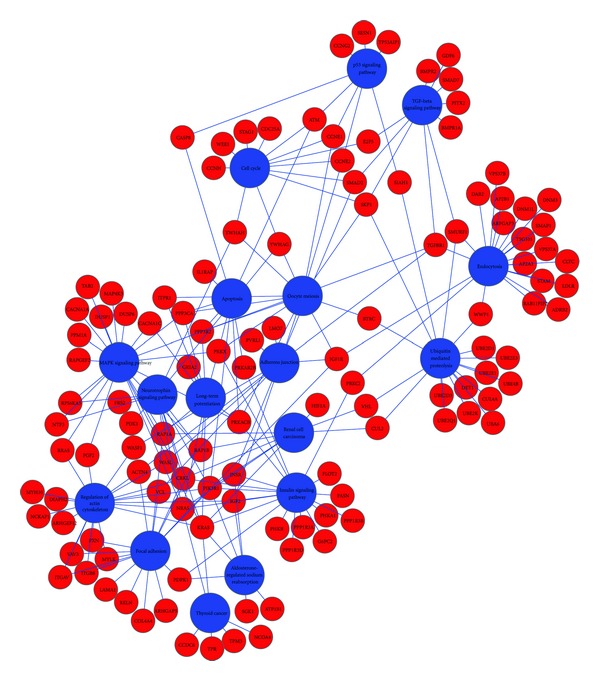
Mapping of miRNA targets genes to biological pathways. This network depicts the miRNA target genes and the associated biological pathways. The landscape of genes to pathway interactions was visualized with Cytoscape. Pink or red nodes represent the genes and the blue nodes indicate biological pathways.

Using the KEGG database we searched for pathways where the miRNA target genes function in order to gain insight into the processes that could be affected by miRNA modulation in irradiated cells. First the mirDB was linked to the Gene Database from the NCBI (National Center for Biotechnology Information) to identify the functions of the specific genes. The Gene Database from the NCBI was retrieved for offline purposes. This database provided descriptive information regarding the functions of miRNA target gene. Second, the Entrez Gene ID was linked to the KEGG pathways database to identify the genes associated with biological pathways. The miRNA target gene dataset was imported into DAVID, a bioinformatics tool that provides functional gene-annotation. The genes associated with various biological pathways were determined with DAVID and the interactions were visualized with Cytoscape (Figure 5). 

The functional pathways highlighted by Cytoscape are shown in Table 3. We were able to map 222 miRNA predicted target genes to 17 biological pathways (Table 3). 22 of these genes were identified in MAPK-signaling pathway, 20 target genes were found to be associated with regulation of actin cytoskeleton, and another 20 genes belonged to endocytosis. A large number of studies have documented the involvement of MAPK-signaling pathway in the response to radiation exposure [33]. The actin cytoskeleton and endocytosis pathways are affected in cells treated with IR [34–36]. We also identified the participation of insulin signaling pathway in radiation response by mapping 19 miRNA target genes to this pathway. The involvement of insulin signaling pathway in radiation response has been reported [37]. Our analysis confirmed the participation of apoptosis, cell cycle, p53 signaling, TGF-beta signaling, all known to be disturbed in cells treated with IR [38–41]. Other pathways that we identified in this analysis and have been documented in radiation response were adherens junction [42], focal adhesion [43], oocyte meiosis [44], renal cell carcinoma [45], thyroid cancer [46], and ubiquitin-mediated proteolysis [47].

Interestingly, the interactome analysis reported in the present study permitted us to discover novel pathways that have not been previously associated with ionizing radiation response. We discovered that radiation-induced miRNA control the expression of a number of genes that function in aldosterone-regulated sodium reabsorption, long-term potentiation, and neurotrophin signaling pathways. 

The mineralocorticoid hormone, aldosterone is a key regulator of sodium homeostasis. The aldosterone controls sodium reabsorption by regulating the cell-surface expression and function of the epithelial sodium channel (ENaC). The stimulatory effect of aldosterone on ENaC is mediated by the induction of serum- and glucocorticoid-regulated kinase 1 (SGK1) [48]. The promyelocytic leukemia zinc finger protein (PLZF) is also upregulated by aldosterone. PLZF is involved in cell cycle control and cell differentiation [49]. How the activation of aldosterone-regulated sodium reabsorption pathway contributes to the response of irradiated cells remain to be investigated.

The neurotrophin-signaling pathway is involved in differentiation and survival of neural cells. The insulin/insulin-like growth factor 1 receptor-signaling (IGF1-R) pathway is linked to the neurogenic capacities of the aging brain, to neurotrophin signaling, and to the molecular pathogenesis of Alzheimer's disease [50]. The response of central nervous system (CNS) to the IR exposure has not been understood. Perhaps signaling molecules act downstream of IGF1-R, and there is a checkpoint to balance excessive growth/“immortality” and reduced growth/“senescence” of a cell. Future investigations might define this connection and its relationship with radiation effects.

The long-term potentiation pathway is associated with long-lasting enhancement in signal transmission between two neurons. The plastic changes at synapses between neurons are partly associated with the memory. The long-term potentiation (LTP) is a major form of synaptic plasticity [51]. The possible implications of long-term potentiation pathway in radiation-induced biological effects remains to be investigated. 

Our findings suggest that the miRNA target gene interactome can help identify novel cellular functions that could be altered as a result of stress induced by radiation exposure. The ability to discover previously uncharacterized new novel pathways through understanding the interactome of miRNA-predicted target genes and -associated pathways offers a new platform for future investigations. A deeper understanding of the miRNA expression signatures in different cell types subjected to IR exposure will not only lead to identify common biological pathways affected in all cell types but will also permit to discover pathways that are only affected in certain cell types.

## 4. Conclusions

It is apparent that miRNAs are involved in controlling the biological pathways associated with ionizing radiation induced stress responses. The miRNA expression alterations in irradiated cells explains the observed biological effects and provides a broader perspective on understanding cellular defense mechanisms against radiation-induced insult. This investigation has provided a starting point where the role of miRNAs in ionizing radiation can be explored. The pathways affected by IR-induced miRNA provide vital information to understand the regulation of the biological processes in cells exposed to IR. It has always been assumed that only transcriptional factors affect the gene expression and control biological pathways. However, the participation of miRNAs adds another set of rules dictating control of the biological pathways. miRNAs may act as “hub” regulators of specific cellular responses, immediately downregulated so as to stimulate DNA repair mechanisms, followed by upregulation involved in suppressing apoptosis for cell survival. Taken together, miRNAs may mediate signaling pathways in sequential fashion in response to radiation.  Future studies will be aimed to understand the effect of miRNA perturbation on the disruption of biological pathways. Though the genes that are associated with the pathways have been determined, it is still unclear whether an activation or inhibition of the pathway takes place in the cells exposed to radiation.

## Figures and Tables

**Table 1:** Radiation-induced miRNA expression analysis in various cell lines.

Cell line	Cell type	Radiation type	Radiation dose (Gy)	Characterized miRNA	Reference
A549	Basal Epithelial	N/A	2.5	8	[11]
HBE135-E6E7	Squamous	N/A	2.5	8	[11]
TK6	Lymphoblast	X-radiation	0.5	21	[13]
TK6	Lymphoblast	X-radiation	2	21	[13]
WTK1	Lymphoblast	X-radiation	0.5	21	[13]
WTK1	Lymphoblast	X-radiation	2	21	[13]
HDMEC	Endothelial	X-radiation	2	11	[15]
AG1522	Fibroblast	X-radiation	0.1	7	[17]
AG1522	Fibroblast	X-radiation	2	22	[17]
M059J	Glial	X-radiation	3	19	[14]
M059K	Glial	X-radiation	3	19	[14]
IM9	B Lymphoblast	Gamma	1	36	[20]
IM9	B Lymphoblast	Gamma	10	26	[20]
IM9	B Lymphoblast	Gamma	0.5	22	[21]
IM9	B Lymphoblast	Gamma	10	22	[21]
TK6	Lymphoblast	Gamma	2	20	[12]
Jurkat	T- cell	Gamma	2	20	[12]
A549	Basal epithelial	Gamma	20	12	[22]
A549	Basal epithelial	Gamma	40	18	[22]
LNCaP	Epithelial	X-radiation	6	15	[18]
C4-2	Epithelial	X-radiation	6	11	[18]
HCT116 (Null)	Epithelial	Gamma	10	36	[19]
HCT116 (WT)	Epithelial	Gamma	10	36	[19]

**Table 2:** miRNA and the predicted target genes identified in biological pathways.

miRNA	Target genes identified in biological pathways
miR-15a, miR-16	AP2A1, PHKA1, CDC25A, SMAD7, CCNE1, SMURF1, PIK3R1, INSR, SESN1, ARHGAP5, UBE4B, YWHAH, SIAH1, BTRC, RELN, UBE2Q1, FGF2, FASN, WEE1, SGK1, TGFBR1
miR-202	SMAP1, DUSP1, LAMA1
miR-155	VAV3, DET1, RAB11FIP, IGF2
miR-197	IL1RAP, ARHGEF12
miR-142-5p	DIAPH2, CUL4A, CCNH, STAG1, RAP1A, UBE2K, TPR, ACTN4, VHL, ATP1B1, ITGAV, CCNG2, UBE2D1, CUL2, HIF1A
miR-142-3p	WASL, MYLK, TAB2
miR-575	GRIA2, TSG101, MYH10
miR-609	PRKC1, PDPK1, PPP1R3A
miR-34A	WASF1, PVRL1, FLOT2, VSP37B, COL4A4, DNM1L, PPM1A, CCNE2, RRAS
miR-34b	YWHAG, DNM3, PDK1, NCKAP1, PRKAR2E
miR-18a	ATM
miR-21	PPP1R3B, NTF3, FRS2, VCL, PPP1R3D, PITX2, UBE2D3, WWP1
miR-let 7a, b, c, d, e, f, g, i	IGF1R, ADRB2, MAP4K3, NRAS, E2F5, GDF6
miR-143	CACNA1A, PRKX, UBE2E1, KRAS, LMO7, UBE2E3, ARFGAP3
miR-376a	TP53AIP1, G8PC2, PRKAC8
miR-19a, miR-19b	RPS6KA5, RAPGEF2, CASP8, VPS37A, CACNA1C, CLTC, ITGB8, LDLR, BMPR2, NCOA4, RAP1B
miR- 145	DAB2, AP2B1, TPM3, SMAD2, DUSP6, PPP3CA, PHHKB, SKP1ITGB8, PPP3R2, CRKL, UBA6, PXN

**Table 3:** Biological pathways controlled by IR-modulated miRNAs and their association with ionizing radiation response.

Identified pathways	Number of miRNA target genes	Association with IR response	Reference
Adherens junction	10	Yes	[42]
Aldosterone-regulated sodium reabsorption	7	Unknown	N/A
Apoptosis	9	Yes	[38]
Cell cycle	12	Yes	[41]
Endocytosis	20	Yes	[35]
Focal adhesion	17	Yes	[43]
Insulin signaling	19	Yes	[37]
Long-term potentiation	11	Unknown	N/A
MAPK signaling	22	Yes	[33]
Neutrotrophin signaling	12	Unknown	N/A
Oocyte meiosis	13	Yes	[44]
p53 signaling	8	Yes	[39]
Regulation of actin cytoskeleton	20	Yes	[34]
Renal cell carcinoma	9	Yes	[45]
TGF-*β* signaling	10	Yes	[40]
Thyroid cancer	6	Yes	[46]
Ubiquitin-mediated proteolysis	17	Yes	[47]
